# Validation of a Mathematical Model for Rupture Status of Spherical Intracranial Aneurysms

**DOI:** 10.1007/s13239-025-00782-1

**Published:** 2025-04-16

**Authors:** Seth Street, Mark D. Johnson, John Na, Paolo Palmisciano, Samer Hoz, Lauren Schaffer, Geet Shukla, Aaron Grossman, Matthew Smith, Peyman Shirani, Jonathan Forbes, Norberto Andaluz, David Dierker, Charles J. Prestigiacomo

**Affiliations:** University of Cincinnati College of Medicine, Cincinnati, USA

**Keywords:** Aneurysm, Biomechanics, Rupture, Cerebrovascular

## Abstract

**Purpose:**

An accurate mathematical model of intracranial aneurysm (IA) mechanics is of great value for its potential utility in assessing probability of IA rupture. Such a model for spherical IAs has been developed which predicts a wall-thickness-to-IA-radius ratio (WTR) of 6.1 × 10^−3^ at which IAs rupture. To our knowledge, no further work has been done to validate this model with clinical data. We aim to assess the accuracy and utility of this model of spherical IA rupture mechanics.

**Methods:**

Aneurysm height, width, neck diameter, and vessel radius were measured on radiologic images of IAs of the basilar terminus, anterior communicating, and posterior communicating arteries. Geometric modeling was used to approximate IA wall thickness. Calculations were performed with and without accounting for changes in IA morphology which have been shown to occur post-rupture. Receiver operating characteristic (ROC) curves and positive likelihood ratios (LR) were produced for WTR, aspect ratio (AR), bottleneck factor (BF), and size ratio (SR). Logistic regression analysis was performed to determine the WTR where there is a 50% chance of presentation as a ruptured aneurysm in our cohort.

**Results:**

52 unruptured and 28 ruptured spherical IAs were included. ROC curve analysis revealed similar areas under the curve for WTR, AR, BF, and SR, ranging from 0.636 to 0.773 with overlapping confidence intervals. LRs ranged from 1.34 (95% CI 1.09–1.65) for AR calculated with post-rupture dimensional adjustments to 2.14 (95% CI 1.45–3.14) for WTR and BF calculated without post-rupture adjustments. Logistic regression revealed a strong association between decreased WTR and rupture status. The point at which there is a 50% chance of presentation as ruptured was found to be WTR = 7.9 × 10^−3^ when calculated without post-rupture adjustments and WTR′ = 6.2 × 10^−3^ when calculated with post-rupture adjustments, from which the proposed 6.1 × 10^−3^ differs by 23% and 1.4%, respectively.

**Conclusions:**

The model for IA rupture mechanics assessed in this study agrees reasonably well with clinical data and could serve as a foundation for further investigation. It additionally performs well in discriminating between ruptured and unruptured aneurysms, though its performance in this dataset is similar to more conventional, simpler parameters. Most importantly, this study demonstrates that biomathematical models can provide valuable insight into the nature of aneurysmal lesions despite their simplifying assumptions.

**Supplementary Information:**

The online version contains supplementary material available at 10.1007/s13239-025-00782-1.

## Introduction

Aneurysmal rupture is an uncommon but serious outcome for patients with intracranial aneurysms (IAs), carrying both high morbidity and high mortality. The current recommendations advise to treat unruptured intracranial aneurysms over 7 mm in diameter [1]. However, numerous observational studies have shown that smaller aneurysms rupture at a non-negligible rate [2, 3]. Treatment for unruptured intracranial aneurysms is not without its own risks. Thus, when making treatment decisions, clinicians require a more accurate metric by which to differentiate aneurysms at higher risk of rupture, thus exposing only those patients to the significant risks of treatment.

In the pursuit of such a metric, some researchers have reported empirical correlations between aneurysm morphology and rupture status, such as aspect ratio (defined as the ratio of aneurysm height to neck diameter), bottleneck factor (defined as the ratio of maximum aneurysm width to neck diameter), and size ratio (defined as the ratio of maximum aneurysm diameter in any direction to parent vessel diameter) [4–6]. These metrics can be useful, but they do not consider the mechanics of aneurysmal rupture and thus ignore potentially important features of IAs, such as wall thickness. Others have chosen to investigate this problem through biomathematical modeling of these lesions. Chaudhry et al. modeled IAs as a thin spherical shell and predicted a critical wall-thickness-to-IA-radius ratio (WTR) of 6.1 × 10^−3^ at which a spherical IA will rupture [7]. Although irregular or multilobular aneurysms are typically of greater concern clinically due to their associated higher rupture risk, modeling IAs as spheres can provide insight on their mechanics [8]. There is some concern that biomathematical models may not provide adequate information due to the numerous assumptions and idealized states applied in them. However, applying a discrete clinical dataset may help to validate some of these models.

In this study, we assess the accuracy and utility of the model proposed by Chaudhry et al. using cases of ruptured and unruptured IAs [7]. Because it has recently been shown by Fujimura et al. that changes in some aneurysm dimensions can occur between pre- and post-rupture states, we perform a second analysis to account for this [9]. We also assess the ability of WTR to discriminate between ruptured and unruptured IAs in comparison to aspect ratio, bottleneck factor, and size ratio. Lastly, we discuss the implications of our findings for future studies.

## Methods

### Patient Selection and Chart Review

After retrospective chart review, 111 patients who presented with IAs of the basilar terminus (BT), anterior communicating artery (Acom), or posterior communicating artery (Pcom) who had computed tomography angiography (CTA), magnetic resonance angiography (MRA), or digital subtraction angiography (DSA) completed between 2005 and 2023 were initially included. These aneurysm locations were selected due to the high frequency of IA initiation and rupture associated with them [10]. Common dichotomized risk factors for IA rupture were also obtained during chart review (history of smoking and history of hypertension). After screening for only spherical aneurysms (defined as not-multilobed and with dimensions that satisfy $\frac{\left|h-w\right|}{h+w}<0.2$), 79 patients were included. Because this research was determined to present no greater than minimal risk, the study protocol received approval from our Institutional Review Board with a waiver of the requirement to obtain informed consent and authorization for the use of protected health information.

### Measurement

**Fig. 1:**
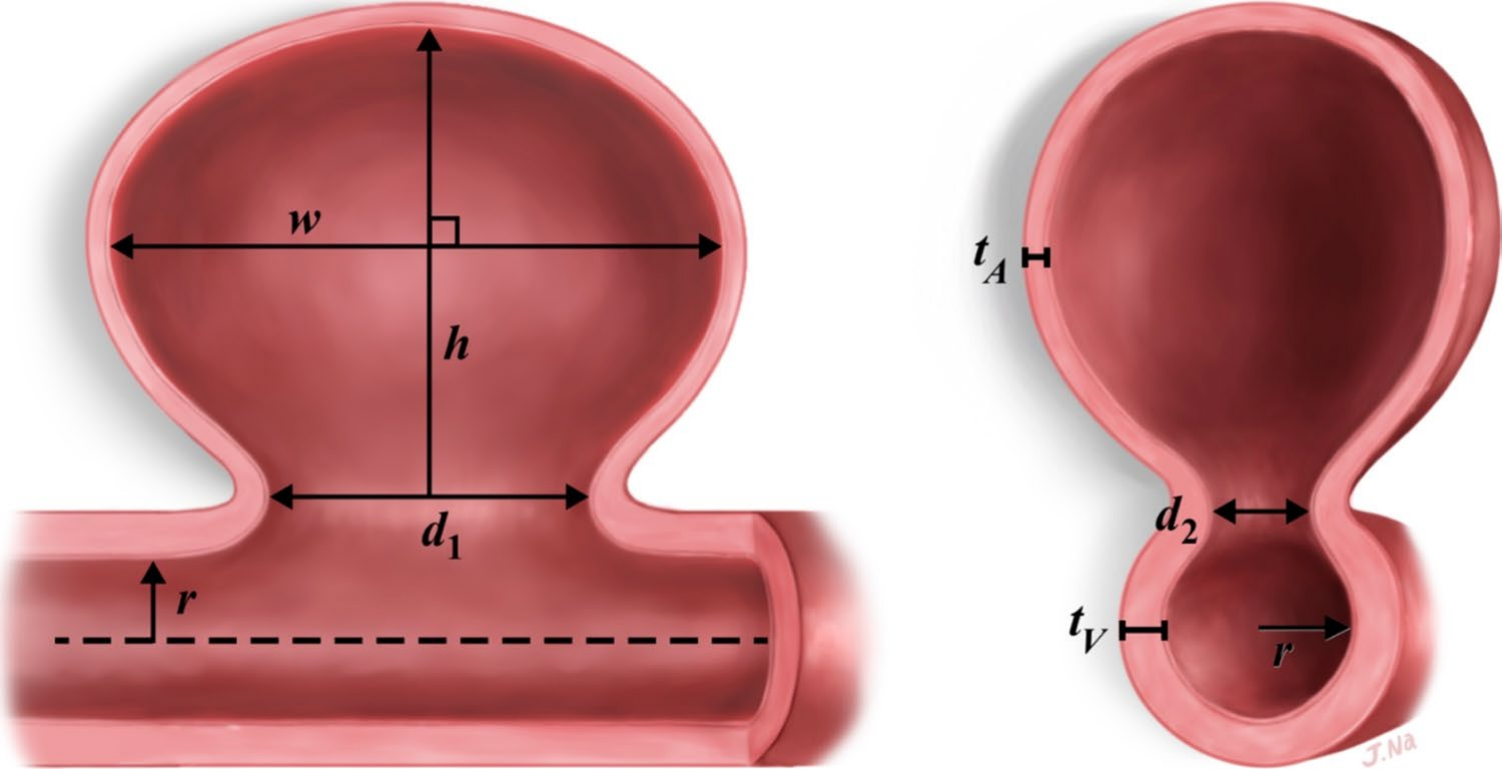
Illustrated definition of variables. The two images are viewed from perpendicular perspectives relative to one another

Measurements of IA height ($h$), width ($w$), parent vessel radius ($r$), and two perpendicular measurements of IA neck diameter (${d}_{1}$, ${d}_{2}$) were made directly on CTAs/MRAs/DSAs. All measurements were made on 2D images. Illustrated definitions of these variables are provided in Figure 1. The parameter, $h$, was defined as the vector of maximum length from the center of the IA neck plane to a point on the aneurysm surface. The parameter, $w$, was defined as the vector of maximum length contained by the aneurysm which was perpendicular to the $h$ vector [5]. The parameters, ${d}_{1}$ and ${d}_{2}$, were defined as the perpendicular vectors of maximum length contained by the IA neck plane. The parameter, $r$, was calculated by dividing the parent vessel diameter just proximal or distal to the IA neck plane, measured perpendicular to the flow vector, by two. For all Acom IAs, the anterior communicating artery was defined as the parent vessel. For BT IAs, the parent vessel was defined as either the basilar artery or the P1 segment of the posterior cerebral artery. If the IA arose entirely from the basilar terminus, the basilar artery was taken as the parent vessel, but if it arose at least partially from the posterior cerebral artery, then the smaller of the two vessels was taken as the parent artery. Likewise, for Pcom IAs, the parent vessel was defined as either the internal carotid artery or the posterior communicating artery, with parent vessel selection proceeding in the same fashion as for BT IAs. This was done because the mathematical model of IA growth used in this study assumes that the surface area of the aneurysm dome is achieved by stretching the parent vessel wall, which means that the thinnest part of the aneurysm dome is that derived from the smaller parent vessel. Therefore, it was assumed that the diameter of the smaller parent vessel primarily contributes to rupture risk.

### Morphometric Details

The wall-thickness-to-IA-radius ratio was defined as

$$WTR=\frac{{t}_{A}}{{R}_{dome}}=\frac{{t}_{A}}{\frac{\sqrt{hw}}{2}},$$

where ${t}_{A}$ is the IA wall thickness and ${R}_{dome}$ is the radius of the IA dome, calculated as the geometric mean of the IA height and width (which approximates the mean diameter) divided by two. The IA wall thickness was approximated as

$${t}_{A}=\frac{S{A}_{neck}}{S{A}_{dome}}{t}_{V}=\frac{\pi \left(\frac{{d}_{1}}{2}\right)\left(\frac{{d}_{2}}{2}\right)}{4\pi {\left(\frac{\sqrt{hw}}{2}\right)}^{2}}{t}_{V}=\frac{{d}_{1}{d}_{2}}{4hw}{t}_{V},$$

where $S{A}_{neck}$ and $S{A}_{dome}$ are the surface areas of the IA neck (modeled as an ellipse) and dome (modeled as a sphere), respectively. The parameter, ${t}_{V}$, is the wall thickness of the parent vessel, which was assumed to be the original thickness of the aneurysm wall before growth and which was approximated as

$${t}_{V}=\left(-0.0297r+0.1187\right)\left(2r\right).$$

This equation was developed using data on the ratio of vessel wall thickness to lumen diameter from Nakagawa et al. to create a linear correlation, as shown in Supplemental Figure 1 [11]. This work was chosen as a reference because its data were obtained through direct intra-operative measurements of cerebrovasculature, which allows for highly accurate in vivo measurements [11]. This is relatively unique among other studies which report similar data, many of which use non-invasive imaging-based techniques to estimate thickness [12]. However, these imaging-based techniques can over-estimate vessel wall thickness due to the presence of artifact [13]. Furthermore, the data provided by Nakagawa et al. match closely with those presented by Prasanna et al., who collected histologic measurements of wall thickness (but did not report on lumen radii) [14].

Because the shape of the aneurysm neck was conceptualized as an ellipse possessing major and minor axes, ${d}_{1}$ and ${d}_{2}$ can be thought of as representing the major and minor axes of the aneurysm neck. The formula $\sqrt{{d}_{1}{d}_{2}}$ was used to calculate the geometric mean of ${d}_{1}$ and ${d}_{2}$, which provides a close approximation of the neck’s average diameter. Thus, aspect ratio (AR), which is the ratio between aneurysm height and neck diameter, was calculated as [14]:

$$AR=\frac{h}{\sqrt{{d}_{1}{d}_{2}}}.$$

Bottleneck factor (BF), which is the ratio between aneurysm width and neck diameter, was calculated as [5]:

$$BF=\frac{w}{\sqrt{{d}_{1}{d}_{2}}}$$

Size ratio (SR), which is the ratio between the maximum length vector contained by the aneurysm dome and the parent vessel diameter, was calculated as [6]:

$$SR=\frac{\text{max}\left(h,w\right)}{2r}$$

In Fujimura et al.’s study comparing pre- and post-rupture IA dimensions, height was significantly greater in post-rupture IAs relative to their pre-rupture state by an average of 14.9% (other dimensions measured in the present study, including IA width and neck size, were unchanged) [9]. To account for this, a second analysis was performed to determine the critical wall-thickness-to-IA-radius ratio (denoted *WTR*′) wherein the measured heights of ruptured IAs were taken to be overestimated by 14.9%, and the corrected height, *h*′, was calculated as

$${h}^{\prime}=\frac{h}{1.149}$$

Using ${h}^{\prime},$ analyses were also repeated for AR and SR, denoted as AR′ and SR′.

### Statistical Analysis

The TRIPOD reporting guidelines were used for this study [15]. Two-tailed heteroscedastic t-tests and chi-squared tests were used to compare the average values of the measured parameters in isolation between ruptured and unruptured cohorts. Receiver operating characteristic (ROC) curves and likelihood ratios were calculated to assess the ability of $WTR$, AR, BF, and SR to discriminate IAs based on ruptured status. Binary logistic regression models were developed to determine the $WTR$ and $WTR^{\prime}$ at which there was a 50% chance of presentation as ruptured in this cohort, and this was compared to the proposed value of 6.1 × 10^−3^. All tests utilized a confidence level of 95% and/or a critical value of $\alpha =0.05$. All statistical analyses were performed in Microsoft Excel v16.92 [16].

## Results

### Data Characteristics

Of the 79 patients included, 51 had unruptured IAs and 28 had ruptured IAs. One patient had two unruptured BT aneurysms, which were two very small (~ 1 mm) posteriorly projecting IAs originating from the basilar terminus. Of the unruptured IAs, 35 were BT aneurysms, nine were Acom aneurysms, and eight were Pcom aneurysms. Of the ruptured IAs, 16 were BT aneurysms, six were Acom aneurysms, and six were Pcom aneurysms. The prevalences of smoking history and hypertension among the ruptured and unruptured cohorts were 80% and 78.75%, respectively (Table 1). Of the 51 included BT IAs, 42 were assigned the basilar artery radius as the parent vessel radius, and nine were assigned a P1 posterior cerebral artery radius. Of the 14 included Pcom IAs, seven were assigned the Pcom radius as the parent vessel radius, and seven were assigned the internal carotid artery radius.

**Table 1:**
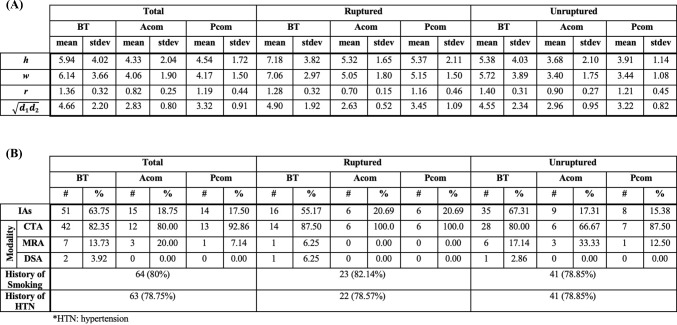
Basic characteristics of collected data (A) continuous variables and (B) discrete variables

There were no significant differences between ruptured and unruptured IAs of the BT, Acom, and Pcom in the average values of $h$, $r$, or $\sqrt{{d}_{1}{d}_{2}}$. However, the average value of $w$ was found to be significantly greater in ruptured Pcom IAs relative to unruptured Pcom IAs (P = 0.042). There was no significant difference in the distribution of IA sites between ruptured and unruptured IAs (P = 0.657). There were also no significant differences in the prevalence of smoking history (P = 0.73) and hypertension (P = 0.98) between the ruptured and unruptured cohorts. Additionally, there were no significant differences in the distribution of imaging modalities between ruptured and unruptured IAs of the BT (P = 0.510), Acom (P = 0.114), or Pcom (P = 0.369) (Supplemental Table 1).

### WTR, AR, and BF Analysis

**Fig. 2:**
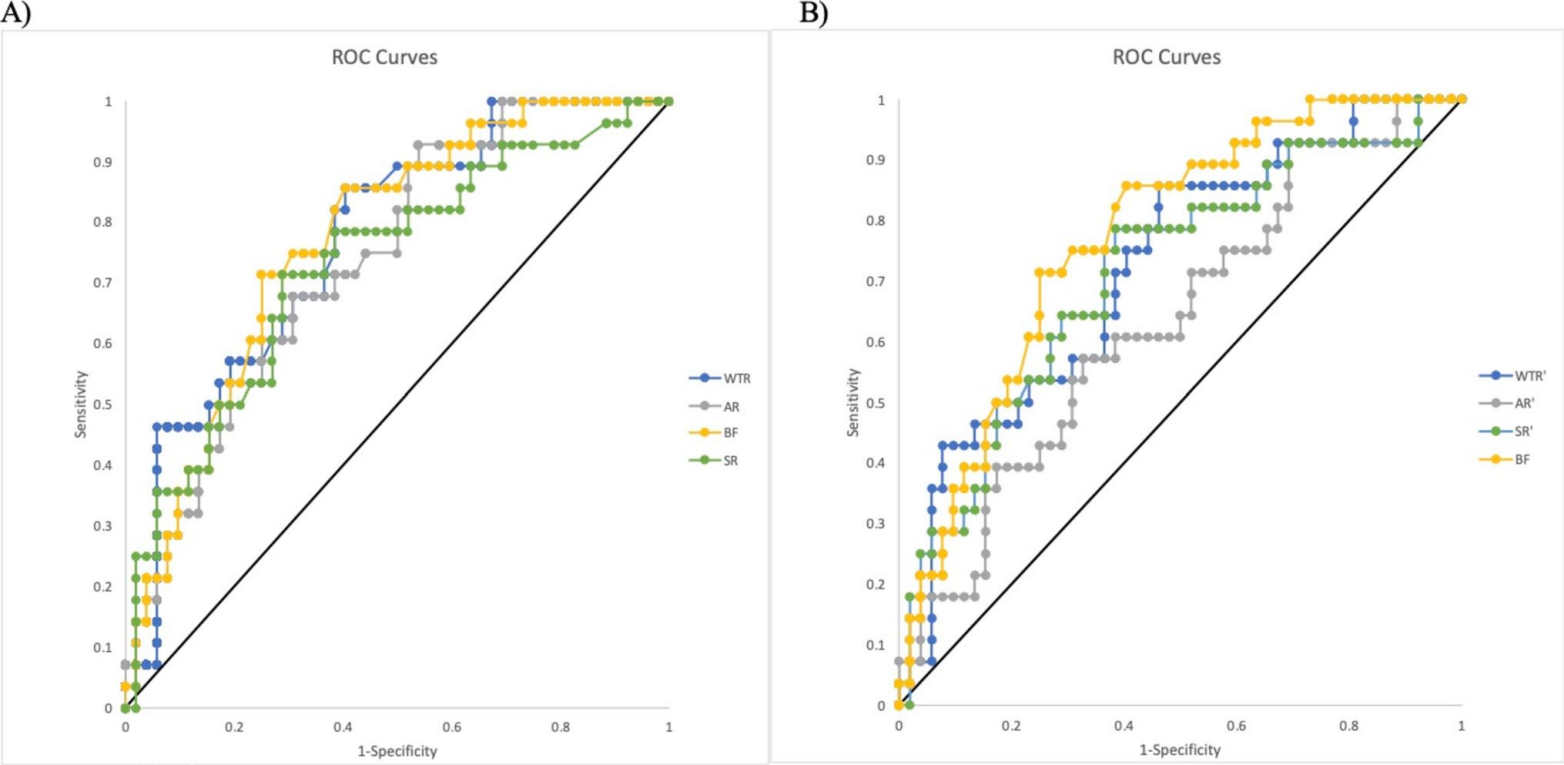
**A** ROC curves for discrimination between ruptured and unruptured spherical lAs based on WTR (AUC = 0.771 [95% CI 0.656–0.885]), AR (AUC = 0.741 [95% CI 0.621–0.860]), BF (AUC = 0.773 [95% CI 0.658–0.887]), and SR (AUC = 0.732 [95% CI 0.611–0.853]. **B** ROC curves for discrimination between ruptured and unruptured spherical lAs based on WTR′ (AUC = 0.724 [95% CI 0.602–0.846]), AR′ (AUC = 0.636 [95% CI 0.505–0.767]), BF (AUC = 0.773 [95% CI 0.658–0.887]), and SR′ (AUC = 0.714 [95% CI 0.591–0.837]

On ROC curve analysis, $WTR$, AR, BF, and SR demonstrated good accuracy when discriminating between ruptured and unruptured spherical IAs, with areas under the curve (AUC) of 0.771 (95% CI 0.656–0.885), 0.741 (95% CI 0.621–0.860), 0.773 (95% CI 0.658–0.887), and 0.732 (95% CI 0.611–0.853), respectively (Fig. 2A). When a cutoff of $WTR=1.49\times {10}^{-2}$ was chosen, $WTR$ discriminated between ruptured and unruptured IAs with 82.1% sensitivity and 61.5% specificity (LR = 2.14 [95% CI, 1.45–3.14]). At cutoffs of AR = 0.990, BF = 1.21, and SR = 1.78, AR yielded a sensitivity of 92.9% and specificity of 46.2% (LR = 1.72 [95% CI, 1.31–2.26]), BF yielded a sensitivity of 82.1% and a specificity of 61.5% (LR = 2.14 [95% CI, 1.45–3.14]), and SR yielded a sensitivity of 82.1% and a specificity of 48.1% (LR = 1.58 [95% CI 1.16–2.16]). When these analyses were repeated using ${h}^{\prime}$, discriminatory performance declined, with $WTR^{\prime}$, AR′, and SR′ having AUCs of 0.724 (95% CI 0.602–0.846), 0.636 (95% CI 0.505–0.767), and 0.714 (95% CI 0.591–0.837), respectively (Fig. 2B). At cutoffs of $WT{R}^{\prime}=1.91\times {10}^{-2}$, AR′ = 0.873, and SR′ = 1.78, $WTR^{\prime}$ yielded a sensitivity of 85.7% and specificity of 53.9% (LR = 1.86 [95% CI 1.33–2.58]), AR′ yielded a sensitivity of 92.9% and specificity of 30.8% (LR = 1.34 [95% CI 1.09–1.65]), and SR′ yielded a sensitivity of 82.1% and specificity of 48.1% (LR = 1.58 [95% CI 1.16–2.16].

**Fig. 3:**
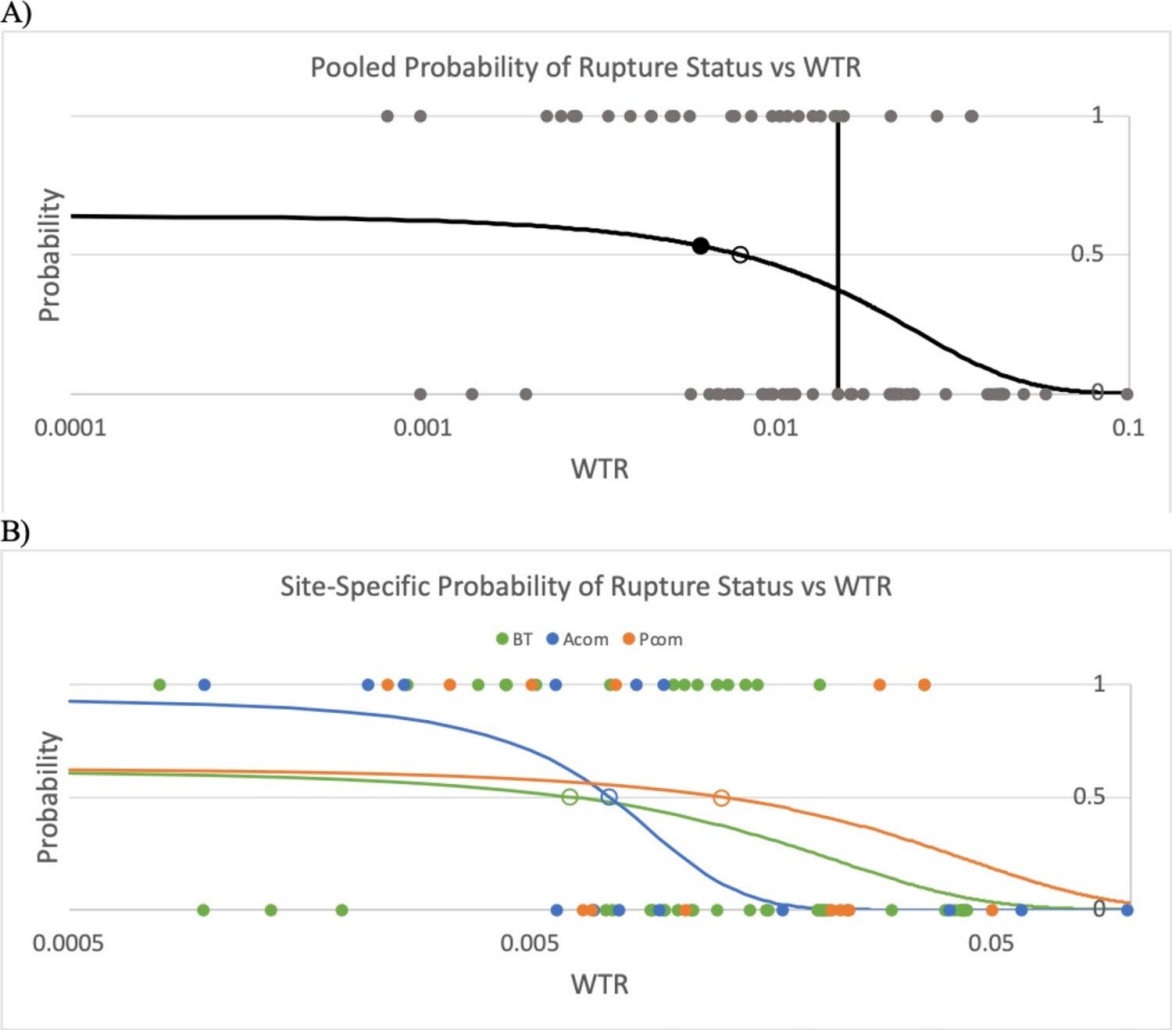
**A** Binary logistic regression model for probability of rupture status across all IA sites as a function of *WTR*. Note that the x-axis is logarithmically scaled. The open circle represents the calculated critical WTR (7.9 × 10^−3^) at which there is a 50% chance of presentation as ruptured. The solid circle is the critical WTR predicted by Chaudhry et al. (6.1 × 10^−3^) [7]. The vertical line represents the cutoff WTR value used in calculating likelihood ratio, sensitivity, and specificity (1.49 × 10^−2^). The function for probability of rupture status is $P(WTR) =\frac{1}{1+{e}^{-0.577+72.8\times WTR}}$ (P = 0.005, OR = 1.08 for every 0.001 increase in *WTR*). **B** Site-specific, color-coded binary logistic regression models for probability of rupture status as a function of *WTR*. The open circles represent site-specific calculated critical WTRs (BT: 6.1 × 10^−3^; Acom: 7.4 × 10^−3^; Pcom: 1.3 × 10^−2^)

**Fig. 4:**
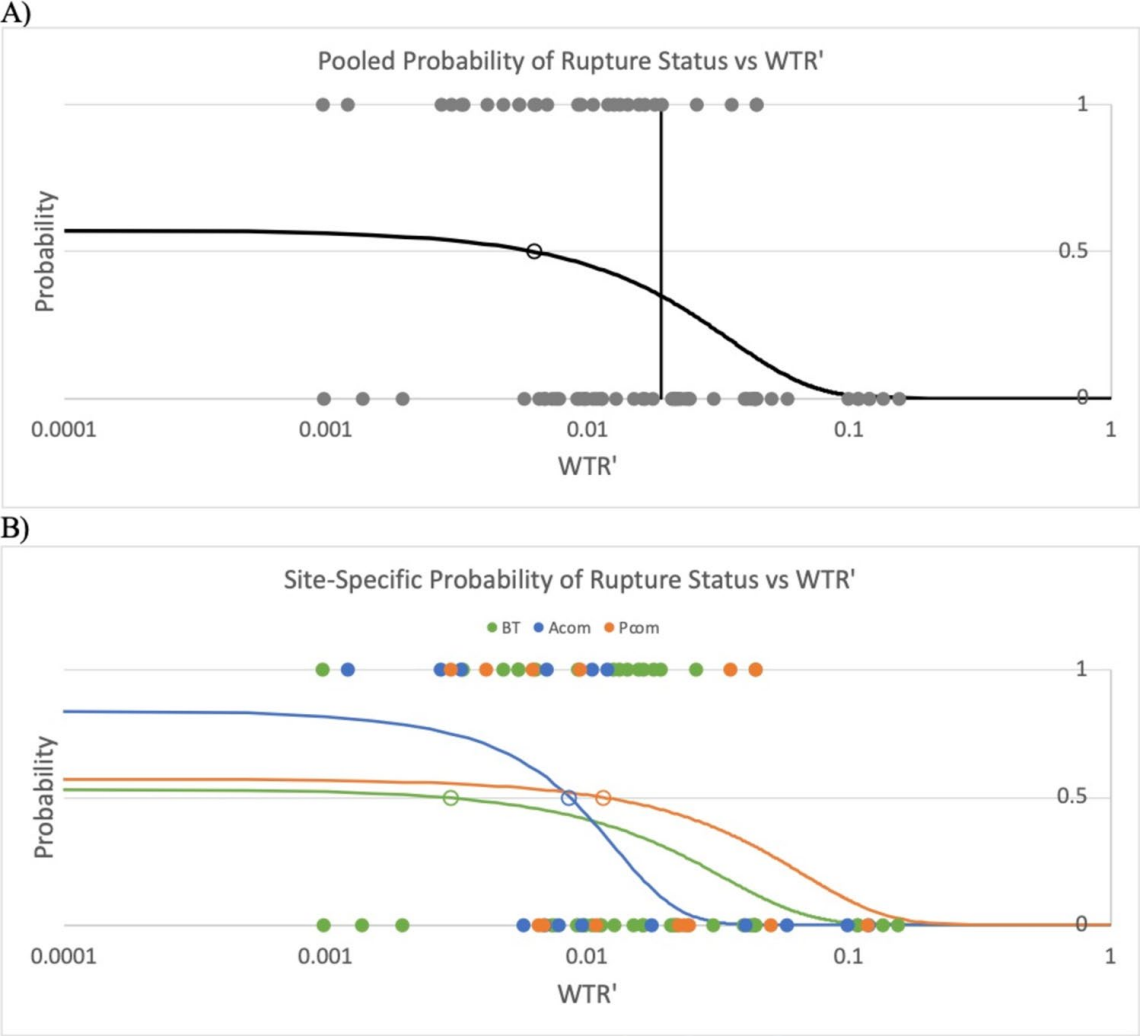
**A** Binary logistic regression model for probability of rupture status across all IA sites as a function of *WTR*′. Note that the x-axis is logarithmically scaled. The open circle represents the calculated critical WTR′ (6.2 × 10^−3^) at which there is a 50% chance of presentation as ruptured, which is very close to the critical value predicted by Chaudhry et al. (6.1 × 10^−3^) [7]. The vertical line represents the cutoff WTR′ value used in calculating likelihood ratio, sensitivity, and specificity (1.91 × 10^−2^). The function for probability of rupture status is $P\left(WT{R}^{\prime}\right)=\frac{1}{1+{e}^{-0.298+48.3\times WT{R}^{\prime}}}$ (P = 0.015, OR = 1.05 for every 0.001 increase in *WTR*′). **B** Site-specific, color-coded binary logistic regression models for probability of rupture status as a function of WTR′. The open circles represent site-specific calculated critical WTRs (BT: 3.0 × 10^−3^; Acom: 8.5 × 10^−3^; Pcom: 1.2 × 10^−2^)

Binary logistic regression analysis also revealed a strong association between decreased $WTR$ and rupture status. The calculated critical $WTR$ corresponding to a 50% probability of presentation as ruptured across all IA sites was found to be $7.9\times {10}^{-3}$, from which the value of $6.1\times {10}^{-3}$ proposed by Chaudhry et al. differs by 23% (Fig. 3A) [7]. Site-specific analysis was also performed, which revealed good agreement in the critical $WTR$ at the BT ($6.1\times {10}^{-3}$) and Acom ($7.4\times {10}^{-3}$), but a larger value was found at the Pcom ($1.3\times {10}^{-2}$) (Figure 3B). When calculated using IA heights corrected for post-rupture changes, the critical $WTR^{\prime}$ calculated across all sites was found to be $6.2\times {10}^{-3}$, from which the value of $6.1\times {10}^{-3}$ proposed by Chaudhry et al. differs by 1.4% (Fig. 4A) [7]. Site-specific analysis of $WTR^{\prime}$ revealed variability in the critical value of $WTR^{\prime}$ by site, with values of $3.0\times {10}^{-3}$, $8.5\times {10}^{-3}$, and $1.2\times {10}^{-2}$ at the BT, Acom, and Pcom, respectively (Fig. 4B).

## Discussion

Our results suggest that the model for spherical IA rupture mechanics proposed by Chaudhry et al. is a reasonably accurate description of wall stress behavior [7]. The theoretical value for $WTR$ at which rupture occurs differs from the calculated result by 23%, and when using corrected values of IA height to account for post-rupture changes, this difference is reduced to 1.4%. Furthermore, using the calculated critical $WTR^{\prime}$ of $6.2\times {10}^{-3}$ to calculate the IA dome wall thickness as ${t}_{A}=WT{R}^{\prime}\times {R}_{dome}$ for a typically-sized IA with ${R}_{dome}=7$ mm reveals a dome wall thickness of ${t}_{A}=43.4$ μm, which is in agreement with most values reported in the literature of 30–200 μm [17]. Additionally, $WTR$ demonstrates a strong ability to discriminate between ruptured and unruptured spherical IAs, with a high AUC (0.771 [95% CI 0.656–0.885]) and LR (2.14 [95% CI 1.45–3.14]). It should be noted, however, that this discriminatory ability is not better than the simpler AR, BF, and SR parameters, whose AUCs and LRs were very similar to those of $WTR$ in this dataset. Lastly, one must recall that this theoretical framework is still limited to only those IAs which are nearly spherical, as this was a fundamental assumption in the derivation provided by Chaudhry et al. [7]. The lack of significant differences in prevalences of smoking and hypertension between the unruptured and ruptured cohorts is likely because these data were not collected from a continuous series of patients but rather from groups of patients pre-stratified by IA rupture status. The similarity of these risk factors between the cohorts likely allowed for better isolation of the effect of $WTR$ on IA rupture.

The discrepancy in calculated critical $WTR$ and $WTR^{\prime}$ values between aneurysm sites suggests that more than just what was considered in Chaudhry et al.’s derivation contributes to rupture risk [7]. For example, the geometry of perianeurysmal vasculature differs by site and can influence wall shear stress experienced inside the aneurysm dome, an important hemodynamic effect which is not considered in the present model [18]. It is also of note that the trend in critical $WTR^{\prime}$ values correspond with the clinically observed risk of rupture at the included sites, with IAs of the Acom and Pcom carrying higher risk than IAs of the BT [19].

Despite this discrepancy, the implications of these results are that the underlying assumptions used in Chaudhry et al.’s mechanical description of spherical IAs are reasonably accurate. These include assumptions that the tension-stretch ratio curve can be modeled by a 2D Fung strain-energy model, that IAs rupture at a magnitude of stress on the order of 1 MPa, and that the mechanics are well-described by large deformation theory [7]. Although it is known that IA wall remodeling occurs over time with IA growth, the results of this study seem to suggest that this remodeling does not have a large effect on the integrity of the wall with regards to rupture risk [20]. Biological tissues have the ability to respond to stress, and it has been observed in histologic studies that collagen deposition occurs non-uniformly in IA walls, likely as a stress response [20, 21]. Because the results of this study align with Chaudhry et al.’s model (which does not consider wall remodeling nor the fact that changes in wall thickness may not be uniform in all axes), the strength of the IA wall appears to be similar to what would be expected without remodeling [7]. The validation of these assumptions provides future theoretical and translational research a solid foundation upon which to build, enabling a more generalized model to be developed which can be applied to the many non-spherical IAs seen in clinical practice. Additionally, these results may have more immediate practical value by allowing neurosurgeons and neurointerventionalists to estimate risk of rupture of spherical IAs by calculating $WTR$ and $WTR^{\prime}$. However, larger sample sizes and prospective validation would need to be employed before concluding that the ability of $WTR$ to discriminate between ruptured and unruptured IAs is truly predictive of IA rupture rather than merely associative with IA rupture-status.

Other authors have offered various mechanistic models of IA rupture with different underlying assumptions. David et al. also examined spherical IAs in their work, but they additionally considered blood pressure to determine risk of rupture at varying IA sizes and blood pressures [22]. Intuitively, their results showed that risk of rupture increases with both IA radius and blood pressure, such that a severely hypertensive patient with a small aneurysm may have comparable risk of rupture to a normotensive patient with a large aneurysm. Although not directly accounted for in the model used by Chaudhry et al., we propose that blood pressure is implicitly considered by the fact that the ratio of IA wall thickness to IA radius will decrease as the IA wall is further stretched, as would happen in the setting of higher blood pressures [7]. Other computational work has shown that IA wall thickness has a large effect on rupture mechanics, further supporting the emphasis placed on this parameter by Chaudhry et al.’s model [7, 23]. These results emphasize a strong relationship between the ratio of IA wall thickness to IA radius which previously has not been as well described and validated with a clinical dataset. This study is also somewhat unique in modeling the aneurysm neck as an ellipse with major and minor axes, a technique which is easy to perform and which improves the accuracy of calculations of the neck area. Furthermore, though histologic studies have provided accurate and objective measurements of IA wall thickness, including varying thickness throughout the wall, this study provides a novel method of estimating IA wall thickness through geometric deduction based on IA radius, which is much more easily measured and is non-invasive [24]. Although it is possible to visualize and measure aneurysm wall thickness directly and non-invasively on imaging using 7 T MRI, this method has its own limitations due to potential overestimation of thickness caused by contrast-induced artifact and due to limited access to these specialized MRI machines [13]. Nevertheless, these data may become very important clinically as imaging technology improves to allow for accessible and accurate non-invasive measurements. Given the significance of aneurysm wall thickness demonstrated in this retrospective validation study and in the theoretical models proposed by other authors, the ability to easily make these measurements could enable accurate stratification of patients by rupture risk

### Limitations

This study was primarily limited by its retrospective nature. Because data were collected retrospectively, it is not possible to say with confidence that the observed discriminative ability of $WTR$ is truly predictive of IA rupture rather than associative. Additionally, because measurements of ruptured IAs were made post-rupture, it is possible that the rupture itself may have altered morphology in such a way as to influence the results, though we attempted to account for this by calculating separately $WTR^{\prime}$. The use of different imaging modalities (CTA, MRA, and DSA) may have introduced noise into our dataset given these modalities have been shown to influence measurements of aneurysmal dimensions differently, though it is unlikely to have biased our statistical analysis as there were no significant differences in the distribution of imaging modalities utilized between ruptured and unruptured aneurysms [25]. This study is also inherently limited due to drawing data from a single center. Some of these limitations include selection bias with the level of lesion complexity that presents to a tertiary/quaternary care center. This referral bias applies to patients who present with both ruptured and unruptured aneurysms. Because there is a tendency to refer more complex lesions, our analysis may have been adversely affected because of a smaller number spherical aneurysms which would present in an unruptured state. Additionally, given data were sourced from just one patient population, there is likely less heterogeneity in smoking status and other lifestyle factors which may affect the biomechanical properties of aneurysms.

The method of approximating wall thickness in this study also has limited accuracy, as it is based on a correlation instead of direct measurement. It is important to note that a fundamental assumption of the model used in this study is that, when an aneurysm arises at least partially from two vessels, it is the vessel of smaller diameter which determines its predisposition to rupture. As discussed in “Methods” section, this decision was made because our model focuses heavily on thinning and thus structural weakening of the IA wall, and we believe it is both biologically and physically intuitive that the wall of the smaller parent vessel would influence this most given that it should possess fewer smooth cells its tunica media at baseline. Furthermore, IA wall thickness is typically not uniform as was assumed in this study and in the study by Chaudhry et al. [7, 21]. Given the non-uniform remodeling which is known to occur in the IA wall, another limiting simplification which must be recognized is the assumption that ${t}_{A}$ can be precisely derived by calculating the extent of deformation endured by the region of parent vessel wall of thickness ${t}_{V}$ from which the IA arose [20]. Finally, this study only analyzed IAs of three sites and notably excluded IAs of the middle cerebral artery. This was done to better assess the strength of association between $WTR$ and rupture status in the setting of high rupture risk. It should also be noted that most of the aneurysms in this dataset are bifurcation-type as opposed to sidewall-type (only three of the unruptured IAs and one of the ruptured IAs were sidewall-type). Studies have shown that sidewall- and bifurcation-type IAs may have distinct forces which drive their initiation and development, with bifurcation-type IAs being influenced more by wall shear stress and sidewall-type IAs being influenced more by impingement forces [26, 27]. While the homogeneity of this dataset does allow for further isolation of the effect of $WTR$, it may threaten the generalizability of this study to sidewall-type IAs. Future studies could improve on these limitations by performing prospective analysis with a greater number of IA sites, including a greater number of sidewall-type IAs.

## Conclusion

The model for IA rupture mechanics proposed by Chaudhry et al. agrees reasonably well with clinical data and could serve as a foundation upon which further investigation may be based [7]. Most importantly, this study demonstrates that biomathematical models can provide valuable insight into the nature of aneurysmal lesions despite their simplifying assumptions. $WTR$ may also prove useful in the future as a tool to predict rupture risk. Although $WTR$ exhibits sensitivity, specificity, and AUC comparable to other tools which are currently used to counsel patients with IAs, such as PHASES, we believe further studies are needed before it is ready for clinical use [28].

## Supplementary Information

Below is the link to the electronic supplementary material.

Supplementary file1 (JPG 185 KB)

Supplementary file2 (JPG 206 KB)

## Data Availability

Anonymized data is available upon reasonable request. If interested, please contact Seth Street (streetss@mail.uc.edu) or Charles J. Prestigiacomo, MD (presticj@ucmail.uc.edu).
