# Supplementary figures and images for: Validation of a Mathematical Model for Rupture Status of Spherical Intracranial Aneurysms
Source: Cardiovasc Eng Technol. 2025 Apr 16;16(4):400–9. doi: 10.1007/s13239-025-00782-1 (PMC12367843; doi:10.1007/s13239-025-00782-1)

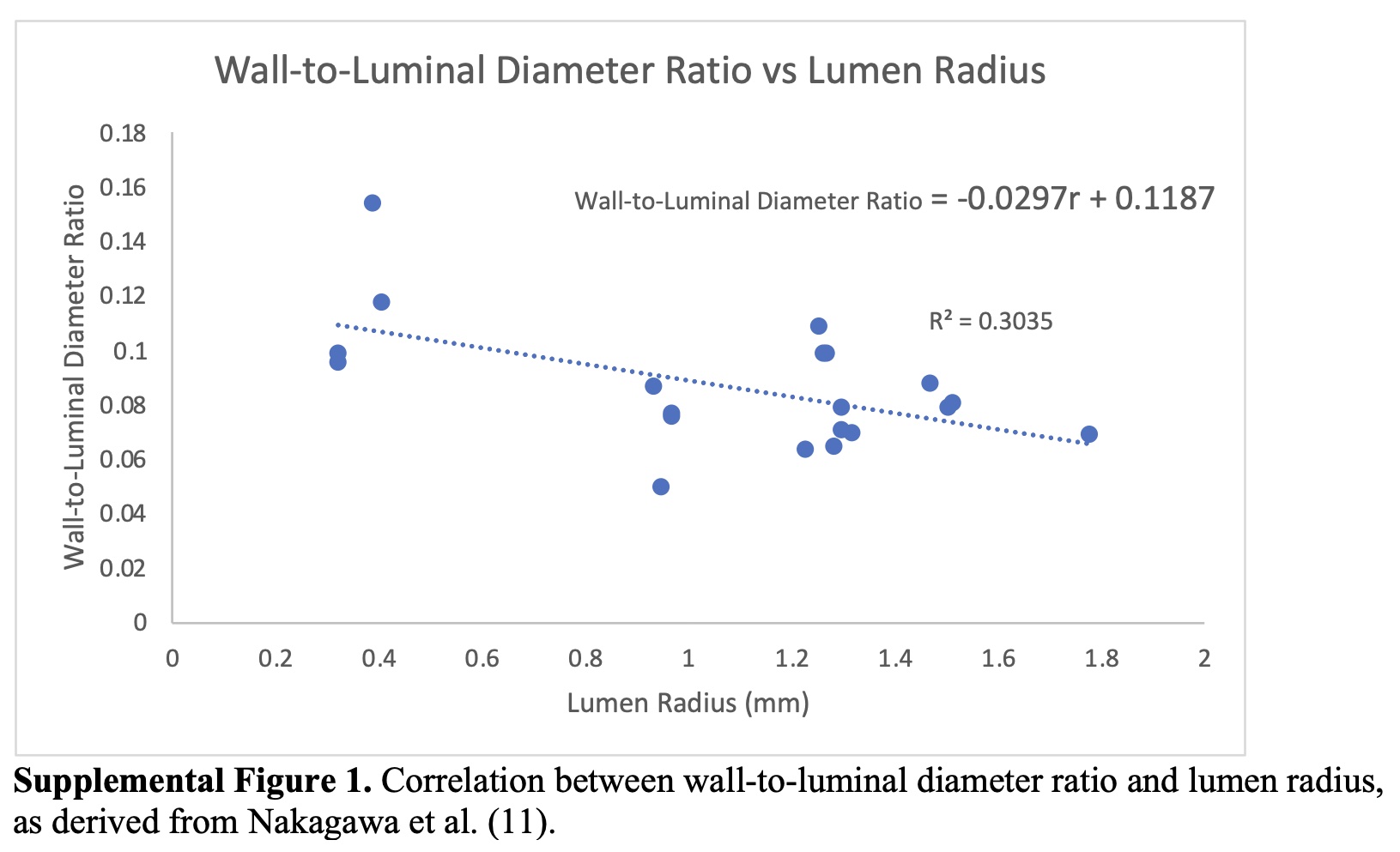

Supplement: Supplementary file 1 — Supplementary file1 (JPG 185 KB) [file 13239_2025_782_MOESM1_ESM.jpg]

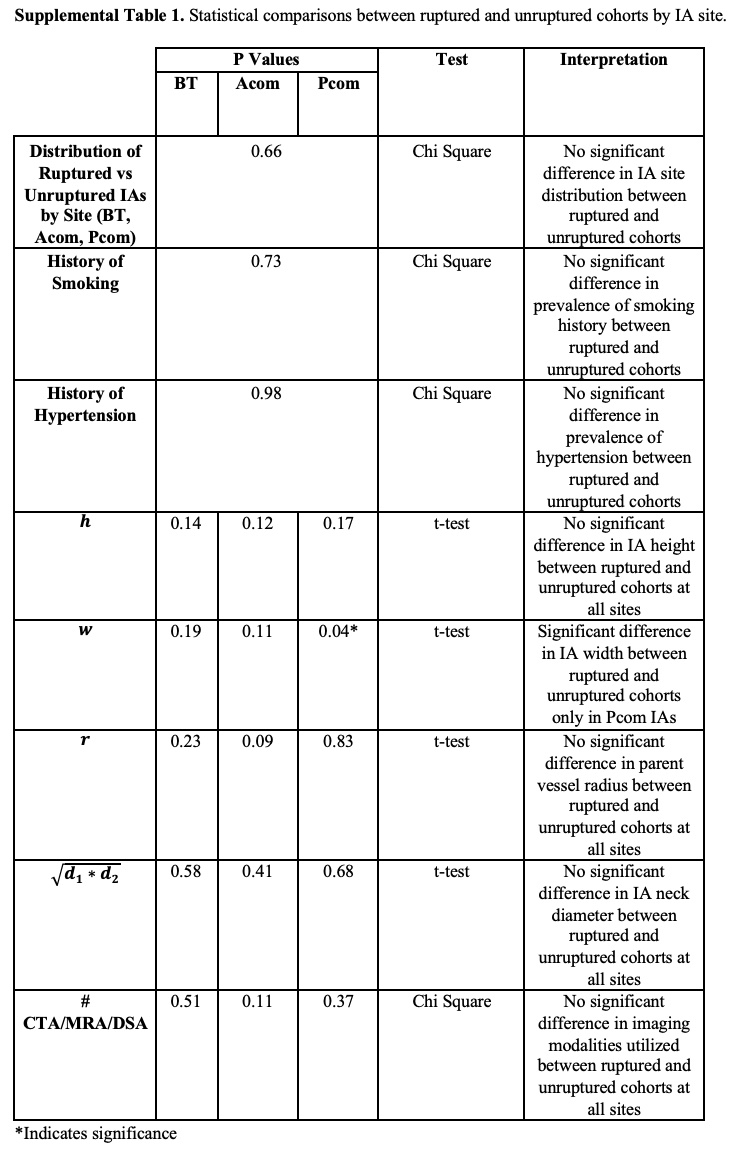

Supplement: Supplementary file 2 — Supplementary file2 (JPG 206 KB) [file 13239_2025_782_MOESM2_ESM.jpg]
